# Physical Inactivity and Food Insecurity Are Associated with Social Capital: A Large-Scale Population-Based Study in Tehran

**DOI:** 10.1155/2022/5410611

**Published:** 2022-11-08

**Authors:** Amin Nakhostin-Ansari, Paria Akbari, Maryam Selk-Ghaffari, Amir Hossein Memari, Mohamad-Reza Vaez-Mahdavi, Mohsen Asadi-Lari

**Affiliations:** ^1^Sports Medicine Research Center, Neuroscience Institute, Tehran University of Medical Sciences, Tehran, Iran; ^2^School of Medicine, Tehran University of Medical Sciences, Tehran, Iran; ^3^Department of Physiology, Medical Faculty, Shahed University, Tehran, Iran; ^4^Immunoregulation Research Center, Shahed University, Tehran, Iran; ^5^Department of Epidemiology, School of Public Health, Iran University of Medical Sciences, Tehran, Iran

## Abstract

**Background:**

There are limited studies on food security, physical activity, and social capital in the Iranian population. This study aimed to evaluate the social capital's associations with physical inactivity and food insecurity in a large-scale study in Iran, Urban HEART-2.

**Methods:**

This cross-sectional study was conducted in 22 districts of Tehran, the capital of Iran. Residents of Tehran who were 15 years or older were selected by a multi-stage, stratified, and random sampling method. Food insecurity and physical activity were evaluated using Household Food Security Scale and Global Physical Activity Questionnaire, respectively, and their associations with social capital were evaluated.

**Results:**

A total of 5030 individuals were included in this study, with 3139 (62.4%) males. The mean age of participants was 44.08 years (SD = 16.33, range = 15–90). Participation in social events (OR = 0.893, 95% CI = 0.819–0.974, *P* = 0.011), social network (OR = 0.849, 95% CI = 0.786, *P* < 0.001), and voluntary activities (OR = 0.865, 95% CI = 0.812–0.921, *P* < 0.001) were all negatively associated with food insecurity. Also, voluntary activities (OR = 0.823, 95% CI = 0.776–0.872, *P* < 0.001) and participation in the associations activities (OR = 0.665, 95% CI = 0.582–0.759, *P* < 0.001) were negatively associated with physical inactivity.

**Conclusion:**

The prevalence of food insecurity and physical inactivity is relatively high among Tehran residents. As a factor affecting the physical activity and food security, social capital can be targeted in interventions to improve physical activity and food security among Iranians.

## 1. Introduction

An unbalanced diet and insufficient physical activity contribute to the increasing burden of chronic diseases in human societies. Food security is known as “physical, social, and economic access to safe food supply with high quality and quantity for everyone to meet their dietary needs and food preferences for an active and healthy life” [1]. Food insecurity is a cause of concern in both developing and developed countries, as it is not only related to the amount of food consumed. A balanced diet that many households might be deprived of in developing and developed countries is another component of food insecurity and is as essential as consumed food [2–4]. Food insecurity is a major public health issue, especially in women, the elderly, and minorities, altering many aspects of daily life, and is associated with a wide range of adverse outcomes on health and quality of life. These adverse outcomes, including obesity, chronic diseases, micro-nutrient deficiency, depression, and fatigue, mostly affect people with lower income or social status [5, 6]. Moreover, the damage of food insecurity to young children's health condition, growth, and development might be considerable and irreversible [7, 8]. Food insecurity, closely related to health inequity, might increase healthcare expenses and consequently put low-income households in an even worse nutrition state [9]. Severe food insecurity is significantly more prevalent in Africa and has a considerably higher index in Asia than Europe [10]. In a study in northwest Iran on 2442 households, the prevalence of food insecurity was 59.3% [11]. Furthermore, based on the Urban HEART-2 project, a population-based study in Tehran, 37.8% of Tehran residents were food-insecure [12].

Physical inactivity is also one of the main preventable causes of mortality worldwide as it is a modifiable risk factor for chronic diseases such as cardiovascular disease, hypertension, type 2 diabetes mellitus, depression, cancers, and obesity [13–16]. Sedentary lifestyle would increase the chance of being overweight and obese, and both have an additive impact on the increasing prevalence of non-communicable diseases in populations [17–19]. Along with unhealthy habits like smoking and environmental risk factors, physical inactivity will eventually lead to higher odds of morbidity and mortality due to preventable causes, which puts a considerable burden on healthcare systems, especially in older ages [20, 21]. Therefore, one of the priorities of health care systems is improving overall physical activity levels among all age groups [22–24]. According to World Health Organization (WHO) recommendations in 2020 guidelines on physical activity and sedentary behavior, all adults must have a weekly physical activity of 150 minutes of moderate or vigorous-intensity aerobic physical activity, on average [25]. In Iran, like the neighbors, the mean physical activity among the urban population is below the average amount recommended by WHO [26, 27]. In a sample from rural and urban areas, 40% had low physical activity levels, and only 32.5% of the urban population had high physical activity levels [27].

Social capital has been a priority in epidemiology and public health research in the 21st century worldwide because of a strong body of evidence supporting its impact on mortality, morbidity, quality of life, general health, and health-related outcomes, including physical inactivity and food insecurity [28–30]. Although various definitions and aspects have been introduced to define social capital, the original definition was brought forward by Bourdieu, which is “the access of individuals and groups to resources facilitated and mediated by their mutual relationships and social cooperation, network, and support,” which provides us with a comprehensive, clear understanding of the term [29, 31]. Evidence from previous studies revealed the positive association between social capital and food security in all groups of people with different social ranks and income levels, especially in high-risk groups like older women [32–34]. According to previous studies, people with higher social capital tend to undertake more physical activity [35–40]. Therefore, interventions focused on increasing social capital in populations may also have beneficial impacts on physical activity levels and food security.

According to limited studies on social capital in Iran, social capital in the Iranian population is not satisfactory, excluding the aspects related to the traditions and culture of ethnic groups. In a large national survey of 12000 individuals, only 26 participants had high social capital [41]. Also, only limited studies in Iran have evaluated the social capital's associations with physical activity levels and food security [42–45]. Therefore, in this study, we aimed to assess the social capital's associations with physical inactivity and food insecurity in a large-scale study in Tehran, the capital of Iran. We hypothesized that better social capital is associated with food security and higher physical activity levels. Policymakers may use the current study's findings to improve social capital, as a possible factor leading to improved physical activity and food security, in their interventions to improve community health. Also, determining the subgroups of social capital that have stronger associations with food security and physical activity may help policymakers design specific interventions.

## 2. Materials and Methods

### 2.1. Design

This cross-sectional study was based on data obtained from a large survey project known as the second round of Urban Health Equity Assessment and Response Tool (Urban HEART-2), conducted in 22 districts of Tehran, the capital of Iran. Originally developed by the World Health Organization (WHO), Urban HEART aimed to generate evidence of inequities in health and its social determinants on local, national, and international scales [46]. The Urban HEART-2 was conducted to assess the general health, social capital, and quality of life of Tehran residents and households selected by a multi-stage, stratified, and random sampling method [47]. In the Urban HEART-2 project, trained interviewers filled out the questionnaires through face-to-face interviews at the door of the selected households.

### 2.2. Study Population

Adult residents of Tehran were included in Urban HEART-2, and data were obtained from raw records of that large multi-aspect survey. There was no limitation in gender, ethnicity, and social class of the ones we included in this study, except that the age limit was at least 15 years old in the original Urban HEART-2 survey. Those whose data regarding social capital, physical activity, and food insecurity were missing were excluded from the study.

### 2.3. Variables

The study population's age, gender, employment status, educational level, marital status, and body mass index (BMI) were also recorded. Participants were divided into five groups in terms of occupation: employed, unemployed, student, retired, and housewife. For this study, we classified educational level as lower than a high school diploma, high school diploma (11 years of education), and higher than a high school diploma. Same as the original study, we put the individuals into single (never married), married, widowed, and divorced categories. BMI was calculated based on the height and weight recorded in the main database by metric units, using the weight (kg)/height^2^ (m^2^) formula. Participants were categorized into three groups based on their BMI: underweight (BMI < 20), normal (20 ≤ BMI < 25), overweight (25 ≤ BMI < 30), and obese (BMI ≥ 30) [48].

### 2.4. Food Insecurity

The Persian version of the Household Food Security Scale was used in Urban HEART-2 study [49, 50]. The questionnaire's sensitivity, specificity, and accuracy were 98.7%, 85.5%, and 89%, respectively [51]. The Household Food Security Scale contains six questions, all in the time interval of the last 30 days. The questions evaluated food shortage or not having enough money to purchase more food, eating limited types of foods due to not having enough money, eating less food than needed due to not having enough money, being hungry but not eating food because of not having enough money, and skipping some meals or decreasing their size due to not having enough money. If the participants answered positively to the last question, they were asked about the number of days it happened over the previous 30 days. Response of three days or more was considered a positive answer. The number of positive answers to questions was calculated, and a score of 2 or more was considered food insecure.

### 2.5. Physical Activity

The Global Physical Activity Questionnaire (GPAQ), an international questionnaire recommended by WHO with confirmed accuracy in previous studies, was used in Urban HEART-2 study to evaluate participants' physical activity levels [52, 53]. Physical activity within a routine week was assessed in three domains: work, travel, and recreation. For each part, the duration and intensity of each activity were assessed, in addition to questions about the days of having vigorous or moderate activities. The aggregated score of physical activity level for each domain was calculated in Mets-hours/week. The level of activity below 600 Mets-hours/week was considered physically inactive.

### 2.6. Social Capital

A valid questionnaire was used in our study to evaluate social capital. This questionnaire was first developed by Abdolahi et al. and is reliable for evaluating social capital with Cronbach's alpha coefficient of 0.88 [41, 54]. The questionnaire consists of nine sections: participation in social events, social network voluntary activities, participation in association activities, social cohesion, trust, social values, view on cultural status, and social support.

The social support section was evaluated by a single question, which could be answered by yes (having social support), no (not having social support), or unsure. Multiple questions evaluated all other sections, and each question could be answered on a Likert scale from 1 to 5. Each section score was calculated by averaging all related items' scores. Higher scores for all these sections were indicative of better social capital status.

### 2.7. Statistical Analysis

We calculated the mean and standard deviation (SD) for continuous variables and the number and percentage for categorical variables. We used chi-squared or Fisher's exact tests to compare the variable in participants based on their physical activity and food security status. Also, we used the Kolmogorov–Smirnov test to determine whether the social capital subscales, age, and BMI values are distributed normally or not. As none of these variables were distributed normally (*P* < 0.05), we used the Mann–Whitney test to compare these variables in participants based on their physical activity and food security status. Finally, we used multiple backward binary logistic regression models to determine the factors independently associated with food insecurity and physical inactivity. We considered *P* ≤ 0.05 as statistically significant. All analyses were performed using SPSS version 22.

## 3. Results

A total of 5030 individuals were included in this study, with 3139 (62.4%) males. The mean age of participants was 44.08 years (SD = 16.33, range = 15–90). Most participants (73.8%) were married, 1927 (38.3%) had an educational level lower than a diploma, and 50.2% were employed. 39.5% of the participants were overweight, and 13.6% were obese. Also, 2846 (56.6%) were physically inactive, and 1714 (34.1%) did not have food security.

Table 1 presents the prevalence of food insecurity and physical inactivity in subgroups. No significant difference between the food security of men and women was reported, whereas men were in a significantly better state of physical activity (*P* < 0.001). A lower educational level was associated with food insecurity and insufficient physical activity (*P* < 0.001). Divorced or widowed people had poorer food security and lower physical activity levels (*P* < 0.001).

Social network (mean = 2.92, SD = 0.76), voluntary activities (mean = 2.88, SD = 1), and social cohesion (mean = 2.74, SD = 0.79) sections had the highest mean scores of eight elements of social capital evaluated in this study.  Table 2 presents the social capital sections' scores based on physical activity levels and food security. The social value section score was not significantly associated with physical activity level and food security (*P* > 0.05). Also, view on cultural status and participation in associations activities sections scores were not significantly different in individuals who had food security and those who had not (*P* = 0.239). Other social capital sections' scores were significantly higher among people with sufficient physical activity levels and those with food security (*P* < 0.05).

Results of multiple binary logistic regressions to determine the factors that were independently associated with food insecurity and physical inactivity are presented in Tables 3 and 4, respectively. Participation in social events (OR = 0.893, 95% CI = 0.819–0.974, *P* = 0.011), social network (OR = 0.849, 95% CI = 0.786, *P* < 0.001), and voluntary activities (OR = 0.865, 95% CI = 0.812–0.921, *P* < 0.001) were all negatively associated with food insecurity. Also, voluntary activities (OR = 0.823, 95% CI = 0.776–0.872, *P* < 0.001) and participation in the associations activities (OR = 0.665, 95% CI = 0.582–0.759, *P* < 0.001) were negatively associated with physical inactivity.

## 4. Discussion

This study investigated the association between social capital, food security, and physical activity in Iran for the first time. Food insecurity prevalence was 34.1% among the study population, and 56.6% of participants did not have the minimum acceptable level of physical activity recommended by WHO. The result demonstrates the significant impact of social capital, especially participation in social events and voluntary activities, on physical activity and food security.

The global prevalence of physical inactivity was reported at 27.5% in 2016 [55]. In the current study, prevalence of physical inactivity was reported at 56.6%, which is considerably higher than global statics. The findings of our study are similar to related studies in Iran. Based on the STEPwise approach to risk factor Surveillance (STEPS) in Iran, the prevalence of insufficient physical activity was reported at 55.4% in 2016 [56]. Food insecurity prevalence was reported at 18.3% in East Asia [57]. The prevalence of food insecurity in the current study was reported at 34.1%, higher than the countries in the region. Hence, major contributors to physical inactivity and food insecurity should be determined for future planning. Physical activity action plans must be developed based on the physical activity facilitators and motivators [58]. Enhancing social capital in Iranian populations could be considered one of the strategies to improve physical activity and food security, considering the positive associations between social capital and higher levels of physical activity and food security. This is especially important in vulnerable groups such as minorities, the elderly, and females. Interventions could be planned to improve social capital and, consequently, increase Iranians' food security and physical activity, which would eventually lead to an increase in physical [59] and mental [60] well-being of individuals, as well as a decline in the prevalence of non-communicable disease and related mortality and complications.

Based on the current study and similar studies in China, Turkey, USA, Canada, and Australia, participation in social events might reduce physical inactivity [35, 36, 61–65]. Social events, which include joining walking groups, sports clubs, or other physical activities, directly decrease physical inactivity. Simultaneously, participating in social events might enhance the availability and dissemination of physical activity health benefit information. Voluntary activities improve knowledge related to the surrounding environments, which are appropriate and encourage physical activity. Consequently, opportunities for physical activity are revealed and improved while participating in social events [61]. In a systematic review by Buja et al., health literacy was proposed as a potential facilitating factor in increasing physical activity [66]. Participating in social events and voluntary activities improves health literacy due to various information sources and facilitates health information exchange [62]. The final consequence may be an improvement in physical activity levels. It should also be noted that engagement in group-based exercise programs may also improve social capital and increase physical activity levels [67], and both physical activity and social capital may affect each other.

Studies about the impact of social capital on food security and physical activity have not been frequent in the Middle East and countries around Iran. Although this was not statistically significant, Sultana et al. reported higher food security in Pakistan's people with better social capital [68]. Also, food security and its association with social capital have mostly been studied in rural societies, as social capital was particularly important in agricultural activities. However, it is important in cities, and a significant association has been found between social capital and food security, especially in poor neighborhoods and slums of big cities [69, 70]. Kirkpatrick and Tarasuk found a significant association between the low social capital of the neighborhood and food insecurity (OR = 1.3) of low-income families in Toronto [71]. Previous studies have indicated the impact of various social capital on food security, particularly bonding and bridging social capital [34, 72–74]. This evidence is compatible with our result, suggesting a relationship between the quality and quantity of nutrition and social capital indicators.

The finding of our study is in line with previous studies, demonstrating that social capital is a determinative indicator in removing barriers to having access to appropriate food security [34]. Having interactive relationships and connecting with the community might facilitate mandated nutritional requirements [34]. Participation in social events, social networks, and voluntary activities were the main domains responsible for decreasing food insecurity in our study. These determinants might increase the knowledge of how to improve the nutritional state of the individuals by enhancing the connection to the community and improving information systems. The available supporting sources and compensatory mechanisms effectively facilitate food security, especially in low socioeconomic populations [34].

The prevalence of physical inactivity and food insecurity is relatively high among Iranian people, which is concerning and requires attention from policymakers [56, 75]. Interventions improving social capital may be beneficial in this regard, as we found that more robust social capital is associated with higher physical activity levels and food security among a sample of Iranian people. Various interventions have been successfully implemented in communities, improving social capital at individual and social levels. Some interventions can enhance both social capital and physical activity levels at the same time [76]. Interventions to enhance social capital among Iranians have been limited. Policymakers can utilize the experiences of other countries in strengthening social capital as a potential intervention to improve physical activity levels and food security among Iranians, and it can be a topic for further studies.

### 4.1. Limitations

This study has a few limitations. First, this study was designed as a cross-sectional study, and we could not evaluate the causal relationship between variables. Second, all measurements were subjective in this study, and future studies may benefit from objective measurements.

## 5. Conclusions

In this study, we found that social capital, especially participation in social events and voluntary activities, might improve physical activity and food security in the setting of Iranian culture. Considering the high prevalence of food insecurity and physical inactivity among Iranians, as indicated in the current study and previous ones, future interventions to improve the Iranian people's lifestyle may benefit from strategies targeting social capital as a possible factor, which can contribute to improving physical activity and food security in Iranian population. This study was cross-sectional, so we could not evaluate the exact interaction between food security, physical activity, and social capital. Therefore, future longitudinal and interventional studies are needed to further assess these factors' associations and design feasible interventions.

## Figures and Tables

**Table 1:** Food security and physical activity among subgroups of the study population.

Variable	Number (%)/mean (SD)	Physically inactive	Physically active	*P* value	Food-insecure	Food-secure	*P* value
Gender							
Male	3139 (62.4%)	1657 (52.8%)	1482 (47.2%)	**<0.001**	1067 (34%)	2072 (66%)	0.878
Female	1891 (37.6%)	1189 (62.9%)	702 (37.1%)		647 (34.2%)	1244 (65.8%)	
Age (years)	44.08 (16.3)	45.56 (16.6)	42.16 (15.7)	**<0.001**	44.07 (16)	44.08 (16.5)	0.646
BMI (kg/m^2^)	25.67 (4.2)	25.78 (4.3)	25.54 (4.1)	0.134	25.89 (4.3)	25.56 (4.2)	**0.012**
Education							
Lower than diploma	1927 (38.3%)	1199 (62.2%)	728 (37.8%)	**<0.001**	864 (44.8%)	1063 (55.2%)	**<0.001**
Diploma (11 years of education)	1489 (29.6%)	796 (53.5%)	693 (46.5%)		508 (34.1%)	981 (65.9%)	
Higher than diploma	1614 (32.1%)	851 (52.7%)	763 (47.3%)		342 (21.2%)	1272 (78.8%)	
Marital status							
Married	3710 (73.8%)	2170 (58.5%)	1540 (41.5%)	**<0.001**	1260 (34%)	2450 (66%)	**0.001**
Single	915 (18.2%)	411 (44.9%)	504 (55.1%)		286 (31.3%)	629 (68.7%)	
Divorced/widowed	405 (8.1%)	265 (65.4%)	140 (34.6%)		168 (41.5%)	237 (58.5%)	
Occupation							
Employed	2526 (50.2%)	1310 (51.9%)	1216 (48.1%)	**<0.001**	818 (32.4%)	1708 (67.6%)	**<0.001**
Unemployed	224 (4.5%)	127 (56.7%)	97 (43.3%)		113 (50.4%)	111 (49.6%)	
Student	277 (5.5%)	129 (46.6%)	148 (53.4%)		92 (33.2%)	185 (66.8%)	
Housewife	1168 (23.2%)	789 (67.6%)	379 (32.4%)		423 (36.2%)	745 (63.8%)	
Retired	835 (16.6%)	491 (58.8%)	344 (41.2%)		268 (32.1%)	567 (67.9%)	

Values are reported as numbers (%), except for BMI and age, which are reported as mean (SD).

**Table 2:** Social capital sections' associations with physical inactivity and food insecurity among the study population.

Variable	Mean (SD)/number (%)	Physically inactive	Physically active	*P* value	Food-insecure	Food-secure	*P* value
Participation in social events	1.98 (0.8)	1.94 (0.7)	2.02 (0.8)	**<0.001**	1.89 (0.7)	2.02 (0.8)	**<0.001**
Social network	2.92 (0.8)	2.89 (0.8)	2.95 (0.8)	**0.008**	2.83 (0.8)	2.97 (0.8)	**<0.001**
Voluntary activities	2.88 (1)	2.79 (1)	3 (1)	**<0.001**	2.73 (1)	2.96 (1)	**<0.001**
Participation in association activities	1.26 (0.4)	1.22 (0.4)	1.31 (0.4)	**<0.001**	1.24 (0.4)	1.27 (0.5)	0.057
Social cohesion	2.74 (0.8)	2.69 (0.8)	2.81 (0.8)	**<0.001**	2.64 (0.8)	2.8 (0.8)	**<0.001**
Trust	2.64 (0.8)	2.61 (0.8)	2.68 (0.7)	**<0.001**	2.54 (0.8)	2.69 (0.7)	**<0.001**
Social values	2.16 (0.7)	2.16 (0.8)	2.17 (0.7)	0.709	2.15 (0.8)	2.17 (0.7)	0.105
View on cultural status	2.65 (0.8)	2.68 (0.8)	2.62 (0.8)	**0.006**	2.67 (0.8)	2.64 (0.8)	0.239
Social support							
No	1533 (30.5%)	917 (59.8%)	616 (40.2%)	**0.008**	575 (37.5%)	958 (62.5%)	**0.002**
Not sure	746 (14.8%)	416 (55.8%)	330 (44.2%)		231 (31%)	515 (69%)	
Yes	2751 (54.7%)	1513 (55%)	1238 (45%)		908 (33%)	1843 (67%)	

Values are reported as mean (SD), except for social support, which is reported as number (%).

**Table 3:** Results of binary logistic regressions to determine the factors associated with food insecurity.

Variable	Odds ratio	95% confidence interval	*P* value
Age (year)	0.995	0.991–0.999	0.01
Educational level of diploma or lower (educational level of higher than a diploma is the reference)	2.502	2.171–2.883	<0.001
Divorced/widowed (being single or married is the reference)	1.290	1.038–1.614	0.022
Unemployment (being employed, student, housewife, or retired is the reference)	2.070	1.568–2.734	<0.001
Participation in social event score	0.893	0.819–0.974	0.011
Social network score	0.849	0.786–0.917	<0.001
Voluntary activity score	0.865	0.812–0.921	<0.001

**Table 4:** Results of binary logistic regressions to determine the factors associated with physical inactivity.

Variable	Odds ratio	95% confidence interval	*P* value
Female gender (male gender was reference)	1.219	1.035–1.435	0.018
Age	1.010	1.006–1.015	<0.001
Single marital status (being married, divorced, or widowed is the reference)	0.766	0.645–0.910	0.002
Housewife (being employed, unemployed, student, or retired is the reference)	1.417	1.166–1.722	<0.001
Voluntary activity score	0.823	0.776–0.872	<0.001
View on the cultural status score	1.101	1.028–1.180	0.006
Participation in association activity score	0.665	0.582–0.759	<0.001

## Data Availability

The datasets used and/or analyzed during the current study are available from the corresponding author on reasonable request.

## References

[1] Simelane K. S., Worth S. (2020). Food and nutrition security theory. *Food and Nutrition Bulletin*.

[2] Van Meijl H., Shutes L., Valin H. (2020). Modelling alternative futures of global food security: insights from Foodsecure. *Global Food Security*.

[3] Yau A., Singh-Lalli H., Forde H., Keeble M., White M., Adams J. (2021). Newspaper coverage of food insecurity in UK, 2016-2019: a multi-method analysis. *BMC Public Health*.

[4] Black R. E., Allen L. H., Bhutta Z. A. (2008). Maternal and child undernutrition: global and regional exposures and health consequences. *The Lancet*.

[5] Gundersen C., Ziliak J. P. (2015). Food insecurity and health outcomes. *Health Affairs*.

[6] Seligman H. K., Laraia B. A., Kushel M. B. (2009). Food insecurity is associated with chronic disease among low-income NHANES participants. *Journal of Nutrition*.

[7] Cook J. T., Frank D. A., Berkowitz C. (2004). Food insecurity is associated with adverse health outcomes among human infants and toddlers. *Journal of Nutrition*.

[8] Shankar P., Chung R., Frank D. A. (2017). Association of food insecurity with children’s behavioral, emotional, and academic outcomes: a systematic review. *Journal of Developmental and Behavioral Pediatrics*.

[9] Berkowitz S. A., Basu S., Meigs J. B., Seligman H. K. (2018). Food insecurity and health care expenditures in the United States, 2011-2013. *Health Services Research*.

[10] Cafiero C., Viviani S., Nord M. (2018). Food security measurement in a global context: the food insecurity experience scale. *Measurement*.

[11] Dastgiri S., Sharafkhani R., Gharaaghaji R., Ghavamzadeh S. (2011). Prevalence, influencing factors and control of food insecurity: a model in the northwest of Iran. *Asia Pacific Journal of Clinical Nutrition*.

[12] Asadi-Lari M., Moosavi Jahromi L., Montazeri A. (2019). Socioeconomic risk factors of household food insecurity and their population attributable risk: a population-based study. *Medical Journal of the Islamic Republic of Iran*.

[13] Rodriguez-Ayllon M., Cadenas-Sanchez C., Estevez-Lopez F. (2019). Role of physical activity and sedentary behavior in the mental health of preschoolers, children and adolescents: a systematic review and meta-analysis. *Sports Medicine*.

[14] Cunningham C., O’ Sullivan R., Caserotti P., Tully M. A. (2020). Consequences of physical inactivity in older adults: a systematic review of reviews and meta-analyses. *Scandinavian Journal of Medicine and Science in Sports*.

[15] Esteghamati A., Abbasi M., Alikhani S. (2008). Prevalence, awareness, treatment, and risk factors associated with hypertension in the Iranian population: the national survey of risk factors for non-communicable diseases of Iran. *American Journal of Hypertension*.

[16] Esteghamati A., Gouya M. M., Abbasi M. (2008). Prevalence of diabetes and impaired fasting glucose in the adult population of Iran: national survey of risk factors for non-communicable diseases of Iran. *Diabetes Care*.

[17] Bista B., Dhimal M., Bhattarai S. (2021). Prevalence of non-communicable diseases risk factors and their determinants: results from STEPS survey 2019, Nepal. *PLoS One*.

[18] Ghimire S., Mishra S. R., Baral B. K. (2019). Non-communicable disease risk factors among older adults aged 60-69 years in Nepal: findings from the STEPS survey 2013. *Journal of Human Hypertension*.

[19] Sithey G., Wen L. M., Dzed L., Li M. (2021). Non-communicable diseases risk factors in Bhutan: a secondary analysis of data from Bhutan’s nationwide STEPS survey 2014. *PLoS One*.

[20] Geidl W., Schlesinger S., Mino E., Miranda L., Pfeifer K. (2020). Dose-response relationship between physical activity and mortality in adults with non-communicable diseases: a systematic review and meta-analysis of prospective observational studies. *International Journal of Behavioral Nutrition and Physical Activity*.

[21] Crosland P., Ananthapavan J., Davison J., Lambert M., Carter R. (2019). The economic cost of preventable disease in Australia: a systematic review of estimates and methods. *Australian and New Zealand Journal of Public Health*.

[22] Tatah L., Mapa-Tassou C., Shung-King M. (2021). Analysis of Cameroon’s sectoral policies on physical activity for noncommunicable disease prevention. *International Journal of Environmental Research and Public Health*.

[23] Duan Y., Shang B., Liang W., Du G., Yang M., Rhodes R. E. (2021). Effects of eHealth-based multiple health behavior change interventions on physical activity, healthy diet, and weight in people with non-communicable diseases: systematic review and meta-analysis. *Journal of Medical Internet Research*.

[24] Reilly J. J., Hughes A. R., Gillespie J., Malden S., Martin A. (2019). Physical activity interventions in early life aimed at reducing later risk of obesity and related non-communicable diseases: a rapid review of systematic reviews. *Obesity Reviews*.

[25] Bull F. C., Al-Ansari S. S., Biddle S. (2020). World Health Organization 2020 guidelines on physical activity and sedentary behaviour. *British Journal of Sports Medicine*.

[26] Rafique I., Saqib M. A., Munir M. A. (2018). Prevalence of risk factors for non-communicable diseases in adults: key findings from the Pakistan STEPS survey. *Eastern Mediterranean Health Journal*.

[27] Esteghamati A., Khalilzadeh O., Rashidi A., Kamgar M., Meysamie A., Abbasi M. (2011). Physical activity in Iran: results of the third national surveillance of risk factors of non-communicable diseases (SuRFNCD-2007). *Journal of Physical Activity and Health*.

[28] Choi S., Oh J., Park S. M. (2020). Association of community level social trust and reciprocity with mortality: a retrospective cohort study. *BMC Public Health*.

[29] Kawachi I. (2006). Commentary: social capital and health: making the connections one step at a time. *International Journal of Epidemiology*.

[30] Clark A., Prætorius T., Torok E., Hvidtfeldt U. A., Hasle P., Rod N. H. (2021). The impact of work-place social capital in hospitals on patient-reported quality of care: a cohort study of 5205 employees and 23, 872 patients in Denmark. *BMC Health Services Research*.

[31] Moore S., Kawachi I. (2017). Twenty years of social capital and health research: a glossary. *Journal of Epidemiology and Community Health*.

[32] Martin K. S., Rogers B. L., Cook J. T., Joseph H. M. (2004). Social capital is associated with decreased risk of hunger. *Social Science and Medicine*.

[33] Lamidi E. O. (2019). Household composition and experiences of food insecurity in Nigeria: the role of social capital, education, and time use. *Food Security*.

[34] Leddy A. M., Whittle H. J., Shieh J., Ramirez C., Ofotokun I., Weiser S. D. (2020). Exploring the role of social capital in managing food insecurity among older women in the United States. *Social Science and Medicine*.

[35] Fu C., Wang C., Yang F., Cui D., Wang Q., Mao Z. (2018). Association between social capital and physical activity among community-dwelling elderly in Wuhan, China. *International Journal of Gerontology*.

[36] Ho E. C., Hawkley L., Dale W., Waite L., Huisingh-Scheetz M. (2018). Social capital predicts accelerometry-measured physical activity among older adults in the US: a cross-sectional study in the National Social Life, Health, and Aging Project. *BMC Public Health*.

[37] Rodrigues D. E., Cesar C. C., Kawachi I., Xavier C. C., Caiaffa W. T., Proietti F. A. (2018). The influence of neighborhood social capital on leisure-time physical activity: a population-based study in Brazil. *Journal of Urban Health*.

[38] Yu C.-Y., Hou S.-I., Miller J. (2018). Health for older adults: the role of social capital and leisure-time physical activity by living arrangements. *Journal of Physical Activity and Health*.

[39] Ganzar L. A. (2019). *Examining the Relation Between Social Capital and Physical Activity Across Life Stages*.

[40] Lindström M. (2011). Social capital, desire to increase physical activity and leisure-time physical activity: a population-based study. *Public Health*.

[41] Abdolahi M., Moosavi M. (2007). Social capital in Iran: current status, prospect, and feasibility. *Social Welfare*.

[42] Koohi K. (2014). Food insecurity and social capital. *Journal of Bioethics*.

[43] Aliyas Z. (2020). Social capital and physical activity level in an urban adult population. *American Journal of Health Education*.

[44] Shiri-Mohammadabad H., Afshani S. A. (2021). Women’s self-care behavior and its relationship with social capital in Yazd. *Iran*.

[45] Rashedi V., Asadi-Lari M., Harouni G. G., Foroughan M., Borhaninejad V., Rudnik A. (2021). The determinants of social capital among Iranian older adults: an ecological study. *Advances in Gerontology*.

[46] Asadi-Lari M. (2010). The application of urban health equity assessment and response tool (Urban HEART) in Tehran concepts and framework. *Medical Journal of the Islamic Republic of Iran*.

[47] Asadi-Lari M., Hassanzadeh J., Torabinia M. (2016). Identifying associated factors with social capital using path analysis: a population-based survey in Tehran, Iran (Urban HEART-2). *Medical Journal of the Islamic Republic of Iran*.

[48] Kabiru W., Denise Raynor B. (2004). Obstetric outcomes associated with increase in BMI category during pregnancy. *American Journal of Obstetrics and Gynecology*.

[49] Blumberg S. J., Bialostosky K., Hamilton W. L., Briefel R. R. (1999). The effectiveness of a short form of the household food security scale. *American Journal of Public Health*.

[50] Gulliford M. C., Nunes C., Rocke B. (2006). The 18 household food security survey items provide valid food security classifications for adults and children in the Caribbean. *BMC Public Health*.

[51] Dastgiri S., Tutunchi H., Ostadrahimi A., Mahboob S. (2007). Sensitivity and specificity of a short questionnaire for food insecurity surveillance in Iran. *Food and Nutrition Bulletin*.

[52] Bull F. C., Maslin T. S., Armstrong T. (2009). Global physical activity questionnaire (GPAQ): nine country reliability and validity study. *Journal of Physical Activity and Health*.

[53] Armstrong T., Bull F. J. (2006). Development of the world health organization global physical activity questionnaire (GPAQ). *Journal of Public Health*.

[54] Kassani A. (2012). Factor analysis of social capital questionnaire used in Urban HEART study in Tehran. *Journal of School of Public Health and Institute of Public Health Research*.

[55] Guthold R., Stevens G. A., Riley L. M., Bull F. C. (2018). Worldwide trends in insufficient physical activity from 2001 to 2016: a pooled analysis of 358 population-based surveys with 1.9 million participants. *Lancet Global Health*.

[56] Kamalian A., Khosravi Shadmani F., Yoosefi M. (2021). A national and sub-national metaregression of the trend of insufficient physical activity among Iranian adults between 2001 and 2016. *Scientific Reports*.

[57] Jones A. D. (2017). Food insecurity and mental health status: a global analysis of 149 countries. *American Journal of Preventive Medicine*.

[58] Memari A.-H. (2021). Action plan to increase physical activity during COVID-19 pandemic. *Sultan Qaboos University Medical Journal [SQUMJ]*.

[59] Rodgers J., Valuev A. V., Hswen Y., Subramanian S. (2019). Social capital and physical health: an updated review of the literature for 2007-2018. *Social Science and Medicine*.

[60] Mehbub Anwar A., Astell-Burt T., Feng X., Feng X. (2019). Does social capital and a healthier lifestyle increase mental health resilience to disability acquisition? Group-based discrete trajectory mixture models of pre-post longitudinal data. *Social Science and Medicine*.

[61] Legh-Jones H., Moore S. (2012). Network social capital, social participation, and physical inactivity in an urban adult population. *Social Science and Medicine*.

[62] Chen W.-L., Zhang C. G., Cui Z. Y. (2019). The impact of social capital on physical activity and nutrition in China: the mediating effect of health literacy. *BMC Public Health*.

[63] Ball K., Cleland V. J., Timperio A. F., Salmon J., Giles-Corti B., Crawford D. A. (2010). Love the neighbour? Associations of social capital and crime with physical activity amongst women. *Social Science and Medicine*.

[64] Yildizer G., Yilmaz I., Novak D. (2019). Social capital and physical activity participation among Turkish adolescents in urban centres: a preliminary study. *South African Journal for Research in Sport, Physical Education and Recreation*.

[65] Yildizer G., Bilgin E., Korur E. N., Novak D., Demirhan G. (2018). The association of various social capital indicators and physical activity participation among Turkish adolescents. *Journal of Sport and Health Science*.

[66] Buja A., Rabensteiner A., Sperotto M. (2020). Health literacy and physical activity: a systematic review. *Journal of Physical Activity and Health*.

[67] Andersen L. L., Poulsen O. M., Sundstrup E. (2015). Effect of physical exercise on workplace social capital: cluster randomized controlled trial. *Scandinavian Journal of Public Health*.

[68] Sultana A., Kiani A. (2011). Determinants of food security at household level in Pakistan. *African Journal of Business Management*.

[69] Obayelu O. A. (2018). *Fod Insecurity in Urban Slums: Evidence from Ibadan Metropolis, Southwest Nigeria*.

[70] Gallaher C. M., Kerr J. M., Njenga M., Karanja N. K., WinklerPrins A. M. G. A. (2013). Urban agriculture, social capital, and food security in the Kibera slums of Nairobi, Kenya. *Agriculture and Human Values*.

[71] Kirkpatrick S. I., Tarasuk V. (2010). Assessing the relevance of neighbourhood characteristics to the household food security of low-income Toronto families. *Public Health Nutrition*.

[72] Shieh J. A., Leddy A. M., Whittle H. J. (2021). Perceived neighborhood-level drivers of food insecurity among aging women in the United States: a qualitative study. *Journal of the Academy of Nutrition and Dietetics*.

[73] Kairiza T., Kembo G., Magadzire V., Chigusiwa L. (2021). Gender gap in the impact of social capital on household food security in Zimbabwe: does spatial proximity matter?. *Review of Economics of the Household*.

[74] Malual J. D., Mazur R. E. (2022). Social capital and food security in post conflict rural Lira District, northern Uganda. *Disasters*.

[75] Behzadifar M. (2016). Prevalence of food insecurity in Iran: a systematic review and meta-analysis. *Prevalence of Food Insecurity in Iran*.

[76] Villalonga-Olives E., Wind T., Kawachi I. (2018). Social capital interventions in public health: a systematic review. *Social Science and Medicine*.

